# Role of FIB-4 for reassessment of hepatic fibrosis burden in referral center

**DOI:** 10.1038/s41598-021-93038-6

**Published:** 2021-06-30

**Authors:** Yun Hwa Roh, Bo-Kyeong Kang, Dae Won Jun, Chul-min Lee, Mimi Kim

**Affiliations:** 1 Department of Radiology, Hanyang University School of Medicine, Hanyang University, Seoul, Korea; 2 Department of Internal Medicine, Hanyang University School of Medicine, Hanyang University, Seoul, Korea

**Keywords:** Diseases, Medical research

## Abstract

Low cut-off of FIB-4 is a widely used formula to exclude advanced liver fibrosis in primary care centers. However, the range of reported threshold of FIB-4 to rule in advanced fibrosis is too broad across etiologies, and no consensus has been reached. In the present study, we investigated the role of FIB-4 for a reassessment of hepatic fibrosis burden in a referral center. We compared the diagnostic performance of FIB-4 among patients with liver disease of various causes and tried to find an optimal cut-off value for predicting advanced fibrosis. Among 1068 patients, the AUROC of FIB-4 to diagnose advanced fibrosis showed no significant difference among the various etiologies of liver disease, ranging from 0.783 to 0.821. The optimal cut-off value obtained by maximizing Youden's index was 2.68, and the sensitivity, specificity, positive predictive value (PPV), and negative predictive value (NPV) for predicting advanced fibrosis were 70.7%, 79.1%, 43.5%, and 92.2%, respectively. The PPV was low in patients with autoimmune disease (6.67%). When we incorporated the new cut-off of FIB-4 into abdominal ultrasound findings, 81% of unnecessary work-ups would be appropriately avoided. In conclusion, the cut-off value of 2.68 showed an acceptable PPV while maintaining a high NPV to predict advanced fibrosis, most etiology except for autoimmune diseases. This result could assist in establishing an appropriate timing to reassess the hepatic fibrosis burden during monitoring in the referral center.

## Introduction

In patients with chronic liver disease, the degree of liver fibrosis is closely related to disease prognosis. Furthermore, compared with healthy controls, the risk of hepatocellular carcinoma (HCC) is 1.36 times higher in patients with significant fibrosis (≥ F2), 2.54 times higher in those with advanced fibrosis (≥ F3), and 5.19 times higher in those with cirrhosis (F4)^1^. The importance of monitoring liver fibrosis is further emphasized in referral hepatic centers that deal with a wide range of underlying diseases.

Liver biopsy is the gold standard for assessing liver fibrosis, but it is invasive and has limited reliability due to inter-observer and intra-observer variations, as well as sampling errors^2–5^. Magnetic resonance elastography (MRE) is an alternative tool that allows quantitative measurement of liver stiffness. Liver stiffness measured on MRE was associated with histological fibrosis progression and the risk of developing clinical liver events^6,7^. However, its utility as a regular examination tool is also restricted due to healthcare costs and accessibility to MRI equipment. Therefore, abdominal ultrasound and laboratory tests are mainly used in the referred center for routine follow-up of chronic liver disease patients.

FIB-4 is the most widely used non-invasive formula for estimating the degree of liver fibrosis. FIB-4 was initially developed to predict significant fibrosis in patients with human immunodeficiency virus and hepatitis C virus (HCV) co-infection^8^. It has been validated in subjects with HCV infection alone, as well as in those with hepatitis B virus (HBV) infection or non-alcoholic fatty liver disease (NAFLD)^9–11^.

A low cut-off of FIB-4 derived from various studies ranges 1.3–1.45, with a high negative predictive value (NPV) of 90%^12^. Accordingly, the low cut-off of FIB-4 is widely used to exclude advanced fibrosis in primary clinics. Meanwhile, the range of reported threshold to diagnose advanced fibrosis as ‘rule-in’ strategy during monitoring chronic liver disease in referral center vague and varies (2.4–10.6) across etiologies^8–11,13^, and no consensus has been reached.

Little is known about a cut-off value of FIB-4 that may be used as a rule-in strategy warranting MRE or liver biopsy in referral centers. In the present study, we compared the diagnostic performance of FIB-4 according to different etiologies. We then tried to investigate a cut-off value that could be used to predict advanced liver fibrosis regardless of etiology.

## Materials and methods

### Study design

The present retrospective cross-sectional study was approved by the institutional review board of Hanyang University Hospital (IRB FILE No.: 2021-02-038-001), and the requirement for written informed consent was waived. All methods were carried out in accordance with the Declaration of Helsinki.

### Inclusion and exclusion criteria

**Figure 1:**
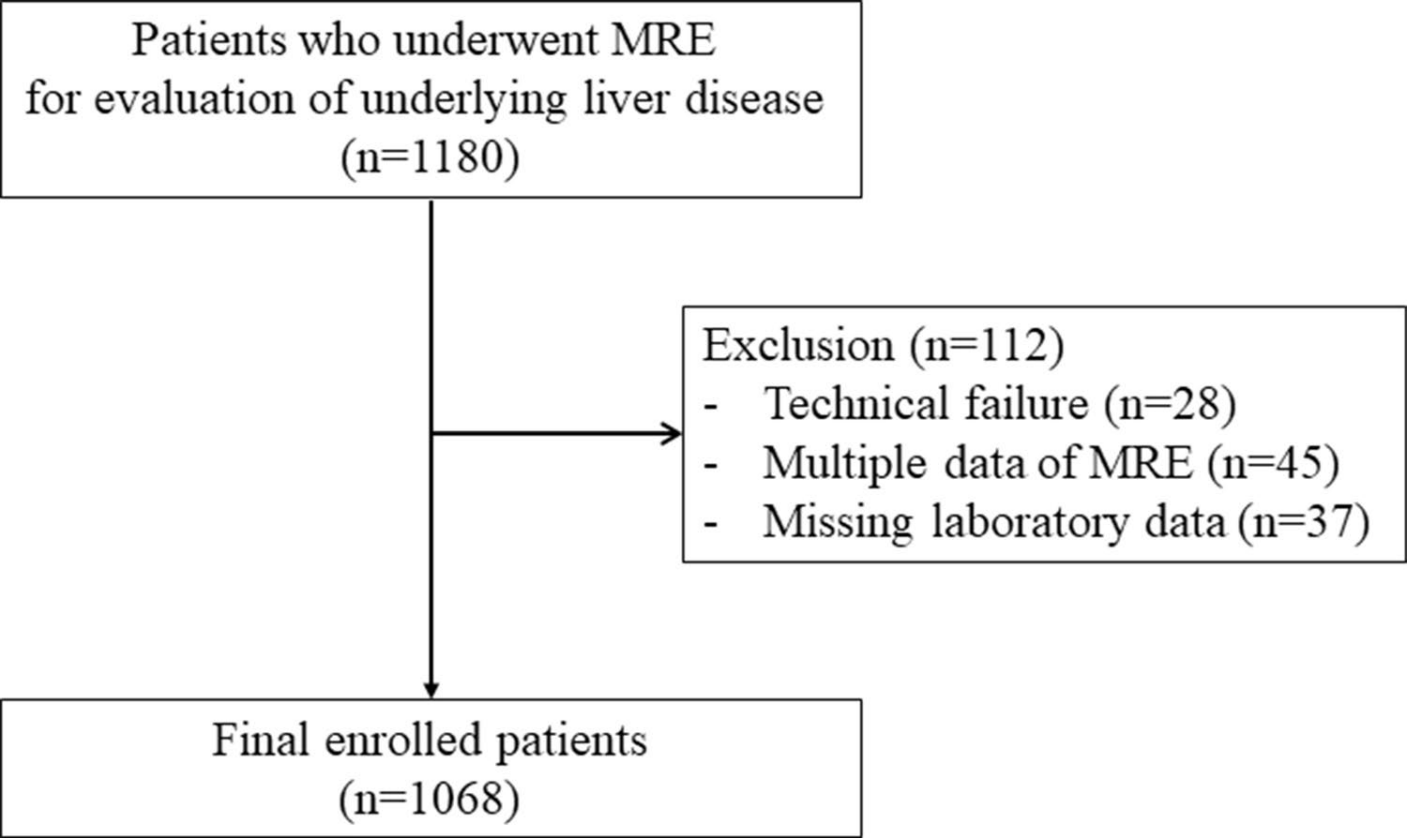
Flow diagram of patient selection, inclusion and exclusion criteria for the study. MRE = MR elastography.

1180 patients who underwent MRE for evaluation of liver disease in a tertiary referral hospital between May 2018 and March 2020 were enrolled in this study. In 45 subjects who underwent MRE more than once (two times, n = 43; three times, n = 2,), the last MRE result was collected. Twenty-eight patients who encountered technical failures during MRE image acquisition were excluded from the study, as were 37 patients who lacked important laboratory data. The final study group comprised 1068 patients (569 men, 499 women; mean age, 54.2 ± 12.4 years) **(**Fig. 1).

### Clinical parameters

Clinical and laboratory data were collected from the electronic medical records of each patient. Clinical data included age, sex, cause of liver disease, presence of HCC, hypertension, diabetes mellitus, and autoimmune disease. Laboratory data closest to the date of MRE were also obtained, including serum levels of aspartate aminotransferase (AST), alanine aminotransferase (ALT), gamma-glutamyl transferase (GGT), total bilirubin, alkaline phosphatase (ALP), albumin, triglyceride (TG), high-density lipoprotein (HDL) cholesterol, low-density lipoprotein (LDL) cholesterol, total cholesterol, and hemoglobin, as well as white blood cell (WBC) count, platelet count, and international normalized ratio (INR). The time interval between the blood test and the MRE was less than 1 week.

### Ultrasonography

Sonographic results taken within 6 months of the MRE were collected. In ultrasound, the degree of liver fibrosis was subjectively assessed by the radiologist on duty and defined as either fibrosis-negative, chronic liver disease, or liver cirrhosis. They were aware that all patients underwent ultrasonography for suspicion of liver disease but were blinded to other clinical or histopathological data. Chronic liver disease was identified when coarsening of the parenchymal echotexture occurred alongside accentuation of the fissure and liver edge blunting, without evidence of surface nodularity. Liver cirrhosis was diagnosed when the ultrasound showed coarse parenchymal echotexture with surface nodularity and signs of portal hypertension, including portosystemic collaterals, splenomegaly, and ascites^12,13^.

### Acquisition and measurement of MRE

MRE examinations were performed using a 3 T magnetic resonance imaging (MRI) machine (Ingenia; Philips Healthcare, Best, Netherlands). Patients were placed in the supine position with a passive driver placed on their right upper abdomen. Continuous low-amplitude 60 Hz vibrations were transmitted to the liver to generate hepatic shear waves, which were imaged using a two-dimensional (2D) gradient-echo (GRE) sequence. Four MRE sections were obtained in each patient. To ensure that the position of the liver was consistent in each section, patients were asked to hold their breath for 16 s after exhalation. The sequence parameters were as follows: 60 Hz mechanical frequency, four phase offsets, axial image plane, superior–inferior sensitizing direction, 50 ms repetition time, 20 ms echo time, 287.4/pixel bandwidth, 30° flip angle, 45 × 40 cm field of view, 300 × 85 matrix size, 10 mm section thickness, and 1 mm intersection gap. Stiffness maps with cross-hatching were generated automatically on the operating console based on the wave amplitude, wave image, and signal-to-noise ratio on the magnitude images. An axial three-dimensional multi-echo modified Dixon GRE sequence (mDIXON-Quant) was obtained simultaneously to evaluate hepatic steatosis, as described in a previous study.

**Figure 2:**
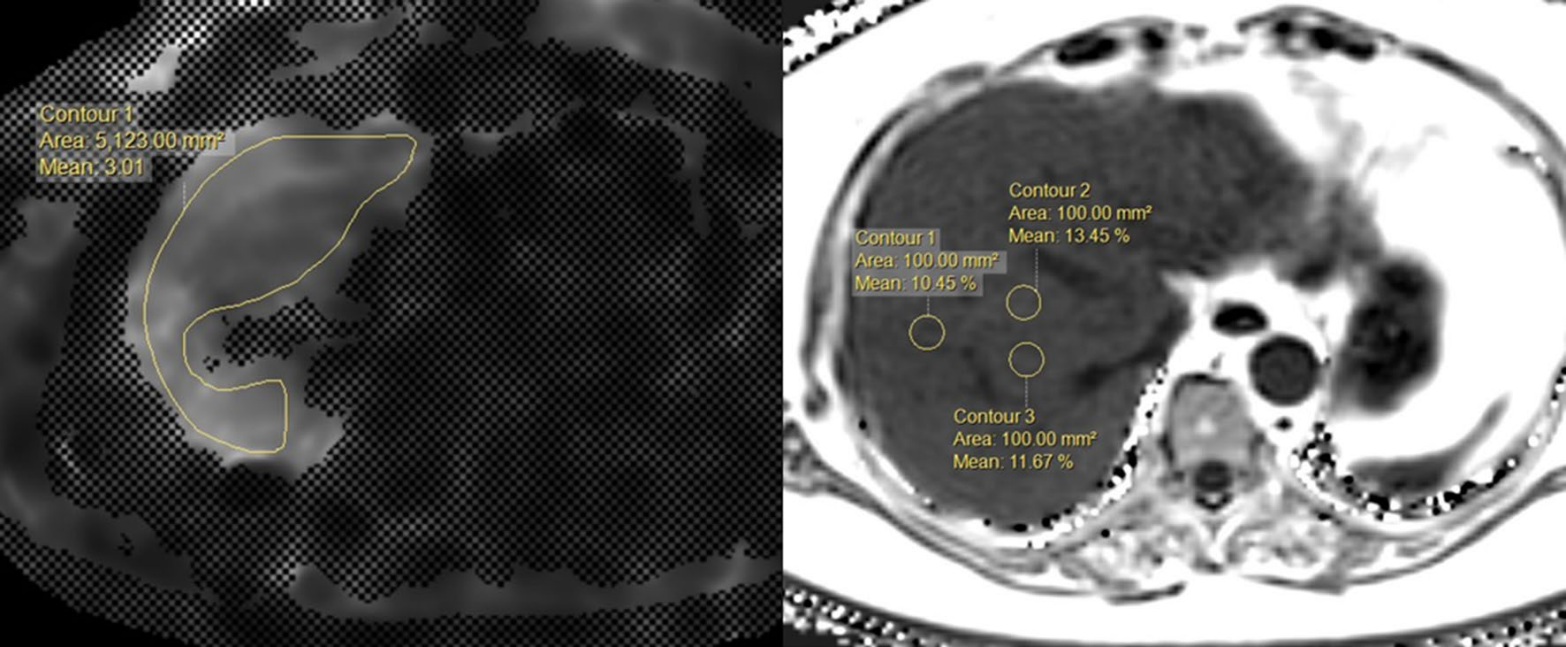
The placement of the ROI for MR stiffness and fat measurement at the workstation. In the MR stiffness map (right), the largest ROI was placed within the contour of the liver and the result are expressed as the mean stiffness (kPa). For the fat quantification (left), three circular ROIs were placed within each Couinaud segment.

Two abdominal radiologists, each with more than 5 years’ experience in reading MRE images, independently measured the liver stiffness values using an MRI software tool (Philips Intellispace Portal 6). They knew that all patients had undergone MRE to assess the possibility of liver disease, but were blinded to the clinical and histological information. In each patient, the region of interest (ROI) was manually placed on a stiffness map of four sections per patient, with reference to the anatomical images. The ROI included the largest part of the liver parenchyma, avoiding voxels with cross-hatching marks, large vessels, liver edge, fissures, and other organs such as the kidney and gallbladder. The average liver stiffness values from the four ROIs of the four sections were used^14^. Inter-observer and intra-observer agreement was 0.991 and 0.995, respectively^14^. The results are expressed as the mean stiffness (kPa), as shown in Fig. 2.

To quantify liver fat, three non-overlapping circular ROIs with an area of 100 mm^2^ were placed within each Couinaud segment, avoiding areas with large vessels, bile ducts, organ boundaries, focal hepatic lesions, or imaging artifacts. In total, 27 ROIs were obtained per patient, and the average of these measurements was considered as the representative hepatic fat fraction of the patient^14^. Liver stiffness and steatosis measurements obtained at the time of examination were entered into the database and extracted for this study. The fibrosis stages were defined as ≥ F2 (significant fibrosis) and ≥ F3 (advanced fibrosis), with thresholds of 3.5 and 4.0 kPa, respectively.

### Non-invasive tests for liver fibrosis

The FIB-4 score, AST-to-platelet ratio index (APRI), and AST/ALT ratio were determined according to previously published formulas^6,15,16^. A level of 40 U/L was defined as the upper limit of normal(ULN) for both ALT and AST levels. The following formulas were used:

$$ \begin{aligned}    & {\text{FIB - 4}} = {\text{Age}}\;\left( {{\text{years}}} \right) \times {\text{AST}}\left( {{\text{U}}/{\text{L}}} \right)/\left[ {{\text{platelet}}\;{\text{count }}\left( {{\text{1}}0^{{\text{9}}} /{\text{L}}} \right) \times {\text{ALT }}\left( {{\text{U}}/{\text{L}}} \right)^{{{\text{1}}/{\text{2}}}} } \right] \\     & {\text{APRI}} = \left[ {\left( {{\text{AST}}\left( {{\text{U}}/{\text{L}}} \right)/{\text{ULN}}\left( {{\text{U}}/{\text{L}}} \right)} \right)/{\text{platelet}}\;{\text{count}}\left( {{\text{1}}0^{{\text{9}}} /{\text{L}}} \right)} \right] \times {\text{1}}00 \\     & {\text{AST}}/{\text{ALT}} = {\text{AST}}\left( {{\text{U}}/{\text{L}}} \right)/{\text{ALT}}\left( {{\text{U}}/{\text{L}}} \right) \\  \end{aligned} $$

### Statistical analysis

Normally distributed numerical variables are presented as mean and standard deviation (SD) and were compared using the independent t-test, and non-normally distributed numerical variables are presented as median (the first quartile–the third quartile) and were compared using the Mann–Whitney test. The correlation between the non-invasive index and MRE was evaluated using Spearman’s correlation test. The MRE values were divided into six cut-offs: 2.5, 3.0, 3.5, 4.0, 4.5, and 5.0, and the diagnostic performance of the non-invasive tests at each cut-off was assessed using the area under the receiver operating characteristic curve (AUROC), with 95% confidence intervals. The AUROC was also calculated for each cause of liver disease, as well as in patients with autoimmune disease. We tried to establish a new single cut-off of FIB-4 using Youden’s index. The diagnostic performance of the new cut-off was assessed using sensitivity, specificity, positive predictive value (PPV), and NPV. The Student's t-test was used to compare the mean values, and the Chi-square test was used to analyze the frequencies of categorical variables. All statistical analyses were conducted using SPSS (version 18.0; SPSS Inc., Chicago, IL, USA) and MedCalc version 17.2 (MedCalc Software, Ostend, Belgium). Statistical significance was set at a *p*-value < 0.05.

## Results

### Baseline characteristics

The baseline characteristics of the patients are summarized in Table 1. A total of 1068 participants were included in the study. The etiologies of liver disease were as follows: alcoholic liver disease (n = 171, 16%), chronic hepatitis B (n = 355, 33.2%), chronic hepatitis C (n = 55, 5.1%), co-infection with HBV and HCV (n = 2), NAFLD (n = 227, 21.3%), other etiologies (n = 258, 24.2%). There were 185 patients with underlying autoimmune disease, 123 with rheumatoid arthritis, 38 with systemic lupus erythematosus, and 24 with other autoimmune diseases such as ankylosing spondylitis, systemic sclerosis, Bechet’s disease, dermatomyositis, mixed connective tissue disease, overlap syndrome, adult-onset still disease, and psoriatic arthritis.

**Table 1:** Baseline characteristics.

Variable	All subjects (n = 1068)	Alcoholic liver disease (n = 171)	Chronic hepatitis B (n = 355)	Chronic hepatitis C (n = 55)	NAFLD (n = 227)	Autoimmune disease (n = 185)
Age, year	56 (47–62)	57 (48–64)	55 (49–62)	60 (56–65)	52 (39–61)	55 (45–61)
Sex, male	569 (51.4)	139 (81.3)	185 (52.1)	24 (43.6)	132 (58.1)	38 (20.5)
AST, IU/L	33 (25–50)	37 (27–62)	29 (24–36)	25 (21–33)	41 (29–64)	38 (29–60)
ALT, IU/L	24 (16–41)	20 (14–32)	22 (17–32)	16 (12–24)	37 (24–67)	26 (17–52.5)
GGT, IU/L	45 (23–91)	89 (45–256)	25 (17–55)	30 (21–47.75)	48 (30–77.25)	38.5 (23–72.5)
Total bilirubin, mg/dL	0.77 (0.61–1.04)	0.95 (0.71–1.47)	0.8 (0.62–1.04)	0.65 (0.55–0.87)	0.75 (0.58–0.95)	0.62 (0.49–0.75)
ALP, IU/L	82 (68–104)	90 (74–124)	80 (66–97.25)	77 (64–96)	79 (67–91)	88 (69–107.5)
Albumin, g/dL	4.3 (4.1–4.5)	4.2 (3.8–4.5)	4.4 (4.2–4.6)	4.3 (4.1–4.5)	4.5 (4.3–4.7)	4.2 (3.9–4.4)
TG, mg/dL	115 (84–167.25)	123 (87–197)	91 (70–137)	108 (81.5–145.5)	142 (112–193.5)	114 (82.5–158.5)
HDL cholesterol, mg/dL	48 (40–58)	45 (30–57.5)	50 (41–61)	44.5 (40.25–58.5)	46 (40–54)	50 (38–62)
LDL cholesterol, mg/dL	100 (80–120.75)	84 (64.5–110)	103 (87–122)	94 (77–110)	107 (86.75–129.25)	103 (85–118.5)
Total cholesterol, mg/dL	178.31 ± 40.16	172.25 ± 45.36	176.89 ± 35.25	171.11 ± 34.93	187 ± 40.63	182.15 ± 38.98
Hemoglobin, g/dL	14 (12.9–15.3)	14.2 (12.4–15.2)	14.4 (13.2–15.5)	13.8 (12.9–15.1)	14.9 (13.8–15.8)	13.3 (12.3–14.2)
WBC (× 10^9^/L)	5.5 (4.5–6.8)	5.3 (4.5–6.7)	5.2 (4.1–6.3)	5.4 (4.6–6.9)	6.6 (5.2–7.6)	5.8 (4.35–7.2)
Platelet (× 10^9^/L)	204 (160.25–248)	181 (119–230)	189 (151–228)	191 (148–228)	235 (198–279)	225 (181–276.5)
INR	1.05 (1–1.11)	1.08 (1.02–1.23)	1.06 (1.02–1.11)	1.05 (1–1.11)	1.03 (0.98–1.07)	1.04 (1–1.1)
Hypertension, n	111 (10.4)	18 (10.5)	31 (8.7)	5 (9.1)	31 (13.7)	19 (10.3)
Diabetes, n	311 (29.1)	65 (38)	71 (20)	18 (32.7)	108 (47.6)	33 (17.8)
Hepatocellular carcinoma, n	39 (3.7)	9 (5.3)	25 (7)	1 (1.8)	0	0
Liver stiffness, kPa	2.5 (2.06–3.38)	3.72 (2.44–5.84)	2.31 (1.95–3.01)	2.63 (2.17–3.65)	2.36 (2.05–2.95)	2.31 (1.94–2.78)
Significant fibrosis (≥ F2), n	252 (23.6)	88 (51.5)	65 (18.3)	16 (29.1)	35 (15.4)	10 (5.4)
Advanced fibrosis (≥ F3), n	198 (18.5)	81 (47.4)	48 (13.5)	10 (18.2)	22 (9.7)	6 (3.2)
PDFF, %	3.5 (2–8.5)	3.3 (2–7.23)	2.6 (1.8–4.3)	2.8 (1.8–4.7)	13.9 (9.5–21.4)	3.2 (1.8–8.5)
**Hepatic fibrosis index**						
FIB-4	1.81 (1.2–3.01)	2.77 (1.62–5.24)	1.81 (1.32–2.67)	1.93 (1.41–3.01)	1.44 (0.94–2.13)	1.7 (1.16–2.67)
APRI	0.42 (0.3–0.71)	0.55 (0.32–1.28)	0.37 (0.3–0.56)	0.35 (0.25–0.5)	0.46 (0.31–0.72)	0.42 (0.29–0.72)
AST/ALT	1.35 (0.96–1.92)	1.88 (1.37–2.69)	1.29 (1.04–1.63)	1.64 (1.1–2)	1 (0.73–1.7)	1.35 (0.97–1.99)
**Ultrasound findings**						
Negative, n	356 (39.1)	34 (22.8)	93 (27.8)	7 (13.2)	119 (79.9)	99 (58.6)
Chronic liver disease, n	294 (32.3)	34 (22.8)	129 (38.5)	29 (54.7)	23 (15.4)	53 (31.4)
Liver cirrhosis, n	260 (28.6)	81 (54.4)	113 (33.7)	17 (32.1)	7 (4.7)	17 (10.1)

Data are presented as mean ± standard deviation, median with interquartile range in parentheses, or numbers of subjects with percentage in parentheses. *NAFLD* non-alcoholic fatty liver disease, *AST* aspartate aminotransferase, *ALT* alanine aminotransferase, *GGT* gamma-glutamyl transferase, *ALP* alkaline phosphatase, *TG* triglyceride, *HDL* high-density lipoprotein cholesterol, *LDL* low-density lipoprotein cholesterol, *WBC* white blood cell count, *INR* international normalized ratio, *kPa* kilopascals, *PDFF* proton density fat fraction.

### Diagnostic performance of FIB-4 in various etiologies

The various liver fibrosis prediction models showed good diagnostic performance (Table 2, Supplementary Fig. S1). The AUROCs of FIB-4 to predict advanced liver fibrosis (≥ F3; 4.0 kPa) and cirrhosis (≥ F4; 5.0 kPa) were 0.821 and 0.85, respectively. The diagnostic performance of FIB-4 at > 4 kPa was superior to that of APRI or AST/ALT. There was a moderate degree of correlation between FIB-4, APRI, and MRE (*r* = 0.494 and *r* = 0.522, respectively), while minimal correlation was noted between AST/ALT and MRE (*r* = 0.224) (Supplementary Fig. S2). The AUROC of FIB-4 to predict advanced fibrosis (≥ F3; 4.0 kPa) was 0.794 in patients with alcoholic liver disease, 0.805 in those with NAFLD, 0.783 in those with chronic hepatitis B, 0.796 in those with chronic hepatitis C, and 0.824 in those with autoimmune disease.

**Table 2:** AUROC of non-invasive hepatic fibrosis score according to various MRE cutoffs.

	MRE ≥ 2.5 kPa	MRE ≥ 3 kPa	MRE ≥ 3.5 kPa	MRE ≥ 4 kPa	MRE ≥ 4.5 kPa	MRE ≥ 5 kPa
**All**						
FIB-4	0.723 (0.693–0.753)	0.780 (0.750–0.811)	0.805 (0.773–0.837)	0.821 (0.786–0.855)	0.817 (0.779–0.855)	0.851 (0.814–0.888)
APRI	0.742 (0.712–0.771)	0.785 (0.755–0.815)	0.813 (0.782–0.844)	0.816 (0.781–0.850)	0.809 (0.771–0.848)	0.839 (0.801–0.878)
AST/ALT	0.585 (0.550–0.619)	0.628 (0.592–0.664)	0.648 (0.607–0.688)	0.676 (0.633–0.719)	0.689 (0.643–0.736)	0.724 (0.673–0.774
**Alcoholic liver disease**						
FIB-4	0.784 (0.70–0.863)	0.822 (0.759–0.885)	0.799 (0.710–0.848)	0.794 (0.728–0.860)	0.761 (0.689–0.833)	0.773 (0.701–0.845)
APRI	0.726 (0.643–0.809)	0.796 (0.727–0.865)	0.786 (0.716–0.856)	0.788 (0.720–0.857)	0.762 (0.688–0.835)	0.768 (0.692–0.844)
AST/ALT	0.715 (0.623–0.807)	0.722 (0.643–0.800)	0.683 (0.604–0.763)	0.707 (0.629–0.785)	0.689 (0.609–0.769)	0.714 (0.636–0.792)
**HBV**						
FIB-4	0.679 (0.623–0.735)	0.715 (0.652–0.779)	0.764 (0.697–0.831)	0.783 (0.704–0.863)	0.826 (0.744–0.907)	0.899 (0.844–0.953)
APRI	0.748 (0.697–0.799)	0.776 (0.720–0.832)	0.840 (0.785–0.895)	0.855 (0.791–0.920)	0.871 (0.792–0.950)	0.935 (0.900–0.970)
AST/ALT	0.456 (0.395–0.517)	0.497 (0.428–0.565)	0.502 (0.421–0.583)	0.520 (0.429–0.610)	0.534 (0.427–0.641)	0.559 (0.435–0.684)
**HCV**						
FIB-4	0.691 (0.552–0.830)	0.754 (0.617–0.891)	0.764 (0.613–0.916)	0.796 (0.615–0.976)	0.763 (0.569–0.957)	0.789 (0.595–0.983)
APRI	0.655 (0.511–0.799)	0.778 (0.645–0.911)	0.829 (0.692–0.967)	0.839 (0.669–1.000)	0.803 (0.619–0.987)	0.845 (0.676–1.000)
AST/ALT	0.583 (0.433–0.733)	0.618 (0.458–0.777)	0.541 (0.350–0.732)	0.549 (0.327–0.771)	0.527 (0.268–0.767)	0.536 (0.251–0.820)
**NAFLD**						
FIB-4	0.692 (0.621–0.763)	0.756 (0.676–0.837)	0.799 (0.708–0.891)	0.805 (0.690–0.920)	0.720 (0.530–0.910)	0.724 (0.476–0.972)
APRI	0.770 (0.709–0.830)	0.803 (0.736–0.869)	0.814 (0.744–0.885)	0.772 (0.681–0.862)	0.731 (0.597–0.864)	0.816 (0.690–0.942)
AST/ALT	0.541 (0.464–0.619)	0.581 (0.487–0.674)	0.661 (0.553–0.770)	0.694 (0.569–0.819)	0.690 (0.521–0.859)	0.659 (0.395–0.923)
**Autoimmune**						
FIB-4	0.628 (0.546–0.709)	0.702 (0.598–0.806)	0.775 (0.628–0.923)	0.821 (0.693–0.949)	0.821 (0.693–0.949)	0.816 (0.661–0.972)
APRI	0.697 (0.620–0.774)	0.772 (0.688–0.856)	0.790 (0.694–0.886)	0.784 (0.676–0.893)	0.784 (0.676–0.893)	0.844 (0.756–0.932)
AST/ALT	0.529 (0.443–0.615)	0.538 (0.428–0.649)	0.676 (0.498–0.854)	0.827 (0.712–0.942)	0.827 (0.712–0.942)	0.811 (0.652–0.969)

*AUROC* area under the receiver operating characteristic; Values in parentheses are 95% confidence interval.

### High cut-off of FIB-4 in referral center in patients with chronic liver disease

The new optimal cut-off based on maximizing Youden’s index was 2.68, similar to the conventional high cut-off of NAFLD (2.67) reported by Shah et al.^17^ (Table 3). The sensitivity, specificity, NPV, and PPV of the new cut-off were 70.7%, 78.9%, 92.2%, and 43.3%, respectively, to predict advanced fibrosis. Table 4 shows the clinical performance of the new single cut-off in various liver diseases. The NPV was low (76.8%) in alcoholic liver disease, while the PPV was considerably low (6.7%) in subjects with autoimmune diseases.

**Table 3:** Determining new cutoff value for FIB-4 in diagnosing advanced fibrosis.

	Cutoff	Youden’s index	Sensitivity (%)	Specificity (%)	PPV (%)	NPV (%)
FIB-4	1.54	0.36	90	46.7	27.7	95.3
	3.53	0.46	56.1	90	55.8	90
	2.68	0.49	70.7	79.1	43.5	92.2

Advanced fibrosis was defined as liver stiffness greater than 4 kPa. *PPV* positive predictive value, *NPV* negative predictive value.

**Table 4:** Diagnostic performance of optimal cutoff value of FIB-4 in diagnosing advanced fibrosis.

Etiology	Cutoff	Sensitivity (%)	Specificity (%)	PPV (%)	NPV (%)
All	> 2.68	70.7	79.1	43.5	92.2
Alcoholic disease		76.5	70	69.7	76.8
Chronic hepatitis B		64.6	81.4	35.2	93.6
Chronic hepatitis C		70	80	43.8	92.3
NAFLD		68.2	85.4	33.3	96.2
Autoimmune disease		50	76.6	6.7	97.9

Advanced fibrosis was defined as liver stiffness greater than 4 kPa. *PPV* positive predictive value, *NPV* negative predictive value.

### Diagnostic performance of high cut-off in old age

Of the 1068 patients, 79.4% (848/1068) were under 65 years old, and 20.6% (220/1068) were over 65 years old. The prevalence of advanced liver fibrosis in < 65 years old was 17.2%, while it was 23.6% in those aged ≥ 65 years. To predict advanced fibrosis, the AUROC of FIB-4 was slightly lower in those over 65 years than in those under 65, but the difference was not statistically significant (0.78, 0.83; *p* = 0.28). The sensitivity, specificity, NPV, and PPV for predicting advanced liver fibrosis in subjects below 65 years old using a FIB-4 cut-off of 2.68 were 66.4%, 85.5%, 92.5%, and 48.7%, respectively. Table 5 shows a comparison of the clinical and laboratory variables based on age.

**Table 5:** Comparison of clinical and laboratory variables based on age.

	Age < 65 (n = 848)	Age ≥ 65 (n = 220)	*p*-value
Age	52 (45–58)	69 (67–72)	
Male, n	441 (52)	107 (48.6)	0.373
AST, IU/L	33 (25–51.75)	32 (25–46.75)	0.49
ALT, IU/L	25 (17–43)	21 (14–29)	< 0.001*
Platelet, (× 10^9^/L)	210.5 (165–254.75)	182 (151–228)	< 0.001*
GGT, IU/L	47 (23–103)	39 (23–73)	0.033*
FIB-4	1.6 (1.1–2.55)	2.93 (2.02–4)	< 0.001*
APRI	0.41 (0.29–0.7)	0.46 (0.33–0.73)	0.097
AST/ALT	1.28 (0.91–1.81)	1.62 (1.15–2.38)	< 0.001*
MRE, kPa	2.42 (2.01–3.3)	2.73 (2.16–3.88)	< 0.001*
Advanced fibrosis, n	146 (17.2)	52 (23.6)	0.029*

Data are presented as median with interquartile range in parentheses, or numbers of subjects with percentage in parentheses. *AST* aspartate aminotransferase, *ALT* alanine aminotransferase, *GGT* gamma-glutamyl transferase, *kPa* kilopascals, *are the parameters with *p* < 0.05.

### Diagnostic performance of abdominal ultrasound

Ultrasound was performed in 910 of the 1068 patients, 356 of whom (33.3%) was normal on ultrasound; 294 (27.5%) showed chronic liver disease and 260 (24.3%) showed liver cirrhosis. There were considerable discrepancies between MRE and sonographic finding. Twenty-six patients (7.3%) showed normal finding on ultrasound, but they had above 4.0 kPa on MRE (advanced hepatic fibrosis). Meanwhile prevalence of advanced liver fibrosis (≥ MRE 4.0 kPa) was only 9.2% (27/294) among subjects with chronic liver disease on ultrasound. The diagnostic performance of abnormal ultrasound findings (chronic liver disease or cirrhosis) to predict the presence of advanced fibrosis was low. Sensitivity, specificity, NPV, and PPV of sonography for predicting advanced liver fibrosis were 85.1%, 44.9%, 92.7%, and 26.9%, respectively.

### Combination of FIB-4 and abdominal ultrasound in referral center

**Figure 3:**
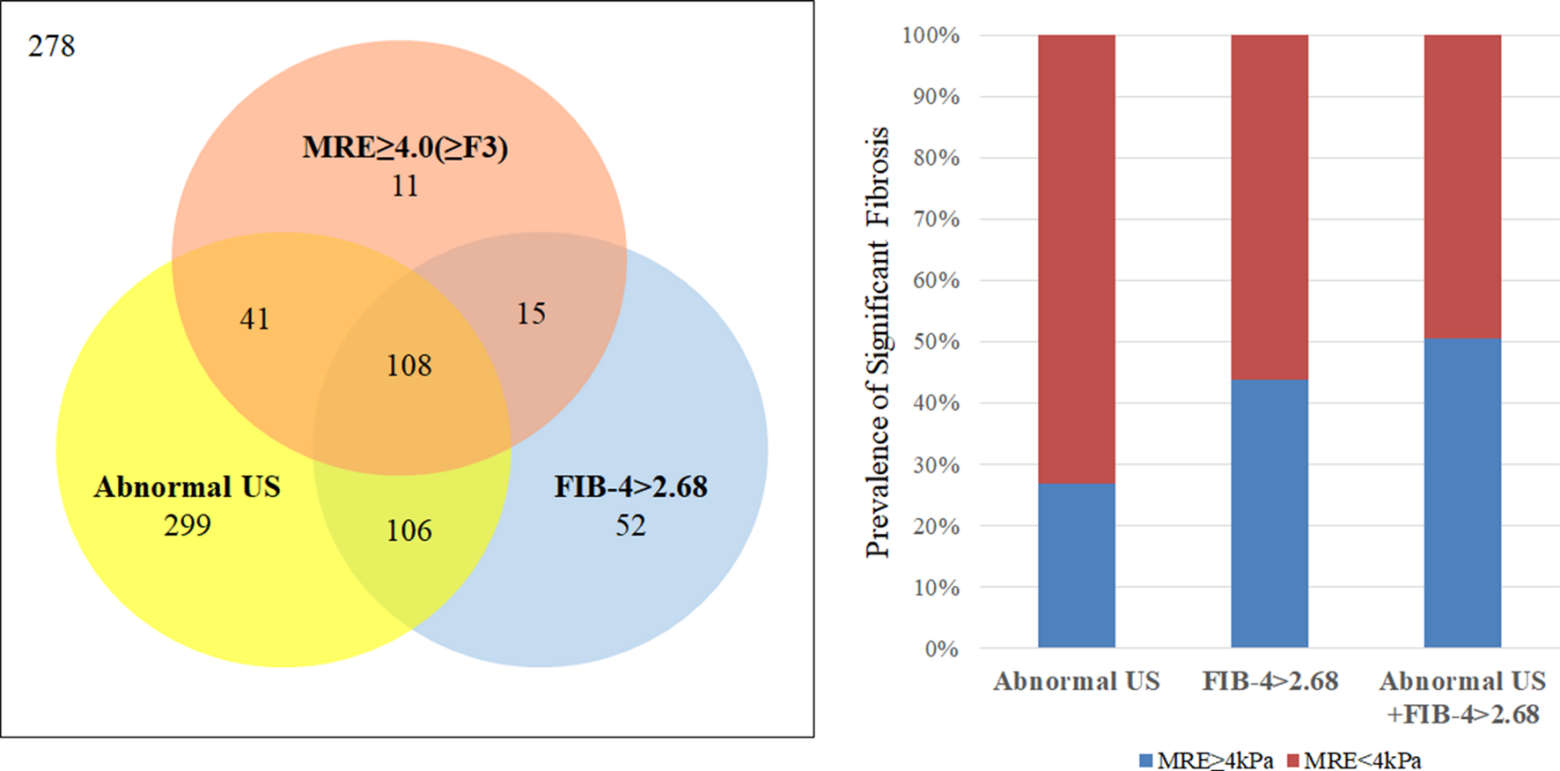
Diagram showing the association of US findings, MRE, and FIB-4. Numbers in circles indicate patient numbers. Abnormal US refers to patients with the sonographic result of chronic liver disease or liver cirrhosis.

The accuracy rate of FIB-4 for diagnosing advanced fibrosis was higher than abdominal ultrasonography (79.6% vs. 52.6%, *p* < 0.05) (Fig. 3). When we combined the new FIB-4 cut-off with abnormal ultrasound findings (sonographic chronic liver disease or cirrhosis patient with FIB-4 > 2.68 is considered to have advanced fibrosis), the accuracy of diagnosing advanced fibrosis increased up to 81.0%.

## Discussion

To the best of our knowledge, this is the first study that evaluated the diagnostic performance of FIB-4 in patients with various etiologies of chronic liver disease. The AUROC of FIB-4 to diagnose advanced fibrosis showed no significant difference among the different etiologies. The optimal cut-off value obtained by maximizing Youden's index was 2.68, which showed acceptable PPV and high NPV regardless of etiology, except for autoimmune diseases.

Abdominal ultrasound is the most commonly used method for monitoring chronic liver disease in a tertiary center (or referral center). Abdominal ultrasound recommended every 6 months in chronic liver disease patients due to the high risk of hepatocellular carcinoma. Although transient elastography or MR elastography frequently used to monitor the progression of hepatic fibrosis, but the optimal timing to perform is unclear. In the long-term management of chronic liver disease, it is necessary to set an optimal timing to re-evaluate the degree of liver fibrosis to predict the prognosis and establish treatment plans. Based on our study, FIB-4 cut-off value (2.68) could identify patients suspected of advanced fibrosis and provide appropriate timing for re-evaluating hepatic fibrosis burden in the referred center. If we incorporate this cut-off value and abdominal ultrasound, we could reduce the number of unnecessary tests that can cause patient discomfort and health care cost.

The optimal cut-off value derived from liver disease of various etiologies was 2.68, which is close to the established high cut-off value of 2.67 reported by Shah et al.^15^. When the cut-off of 2.68 was applied, the sensitivity, specificity, NPV, and PPV to predict advanced fibrosis among all subjects were 70.7%, 78.9%, 92.2%, and 43.3%, respectively. The PPV was highest among patients with underlying alcoholic liver disease (69.66%). Predictive values can be expressed as the probability that the test result is correct; such values are affected by the prevalence of a disease in a population^16^. Therefore, the PPV of FIB-4 in alcoholic liver disease was likely high because advanced fibrosis was more prevalent in our population (47.4%) than other etiologies. The PPV was low (6.67) in patients with autoimmune disease, perhaps because of changes in platelet count that result from chronic inflammation in autoimmune diseases. So we added and modified expression regarding ‘sing cut-off’ in different liver disease etiologies.

Although our new cut-off was derived from various chronic liver diseases, the diagnostic performance of FIB-4 in the present study was comparable to that of previous results. Shah et al.^15^ examined 541 subjects with NAFLD in whom the prevalence of advanced fibrosis was 23%; they proposed a high cut-off value of 2.67, which showed a sensitivity, specificity, NPV, and PPV of 33%, 98%, 83%, and 80%, respectively. Kim et al.^9^ evaluated the diagnostic value of FIB-4 in 668 patients with chronic hepatitis B in whom the prevalence of F3–4 was 49.4%; they suggested an optimized cut-off value of 2.65 to predict severe fibrosis (F3). The sensitivity, specificity, NPV, and PPV were 38.5%, 97.9%, 61.9%, and 94.8%, respectively.

The diagnostic accuracy of FIB-4 varies with age^17–19^, and since age is one of the variables of the FIB-4 formula, the value of FIB-4 increases in older patients. In the present study, the specificity and PPV of FIB-4 were significantly lower in the older age group, probably because FIB-4 has a higher value in these patients, resulting in higher false-positive rates. For the above reasons, a different low cut-off (2.0) should be used in those over 65 years old^17^. However, no research has addressed whether other high cut-offs should be used. In the present study, the AUROC of FIB-4 was higher in patients aged < 65 years than in those aged > 65 years, although the difference was not statistically significant (0.832 vs. 0.782).

Liver ultrasound is a surveillance tool in patients with chronic liver disease. Although ultrasound shows low sensitivity and specificity to detect fibrosis, it is cost-effective and useful for monitoring HCC^20^. When we combined the new FIB-4 cut-off with abnormal ultrasound findings (sonographic chronic liver disease or liver cirrhosis with FIB-4 > 2.68, which is considered to indicate advanced fibrosis), the accuracy of diagnosing advanced fibrosis was higher than when using FIB-4 or sonographic results alone (Fig. 3). Assuming that an unnecessary additional work-up could be avoided in patients who had a true negative or true positive test result, 81% of unnecessary work-ups would be avoided by incorporating FIB-4 and ultrasound results to evaluate subjects with advanced fibrosis.

We acknowledge that the present study was limited because we did not use liver biopsy to diagnose the stages of liver fibrosis. Although liver biopsy is an imperfect tool, it is still the gold standard. However, several studies have reported the diagnostic accuracy of MRE in staging fibrosis and that it could be used as an alternative to liver biopsy^21^. We believe that the fibrosis staging derived from MRE in the present study would not have differed from the results of a liver biopsy. Second, medication such as antiviral agent that can affect liver fibrosis prescribed if necessary, for ethical reasons until you collected the blood samples to calculate FIB-4 score and MRE examinations. MRE and laboratory tests were conducted within a week. It is unlikely that a week of (antiviral) treatment will affect the stage of liver fibrosis. However, sonography performed within 6 months from the MRE examination. Six months treatment including antiviral treatment might affect their stage of hepatic fibrosis. The results should be interpreted with caution due to the study limitations.

In summary, the cut-off value of FIB-4 (2.68) showed acceptable PPV while maintaining high NPV in predicting advanced fibrosis in various etiologies in a referred center. Incorporation of the present cut-off value with sonographic result increases diagnostic accuracy for ruling in patients with advanced fibrosis. The result of present study could assist in establishing an optimal timing to re-evaluate the degree of liver fibrosis and set treatment plans in the referral center.

## Supplementary Information

Supplementary Figures.
