# Supplementary material for: Role of FIB-4 for reassessment of hepatic fibrosis burden in referral center
Source: Sci Rep. 2021 Jun 30;11:13616. doi: 10.1038/s41598-021-93038-6 (PMC8245508; doi:10.1038/s41598-021-93038-6)
Supplement: Supplementary file 1 — Supplementary Figures. [file 41598_2021_93038_MOESM1_ESM.pdf]

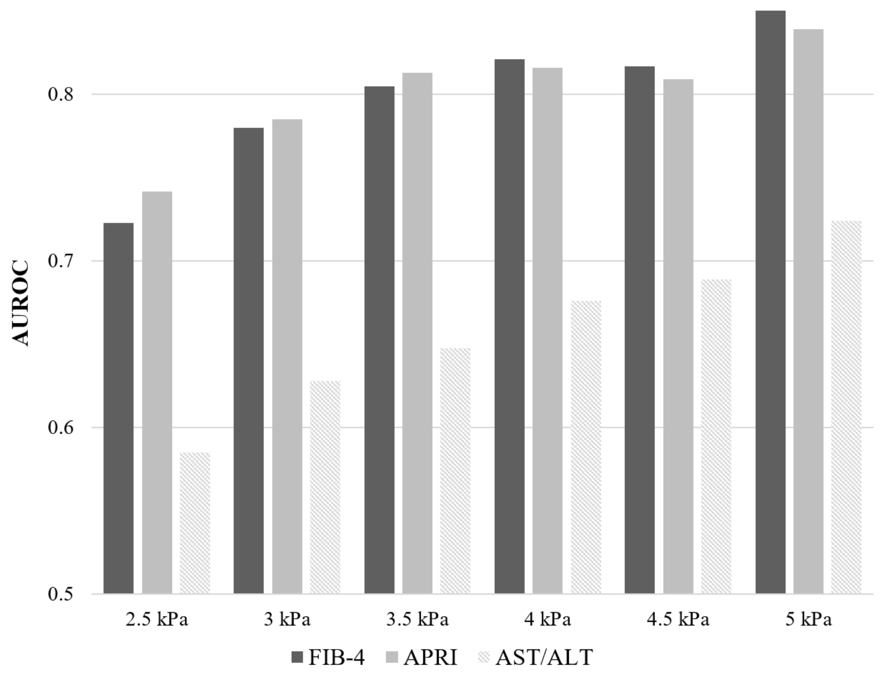

Supplementary Figure S1. Comparison of AUROC of hepatic fibrosis index according to various MRE cut-offs.

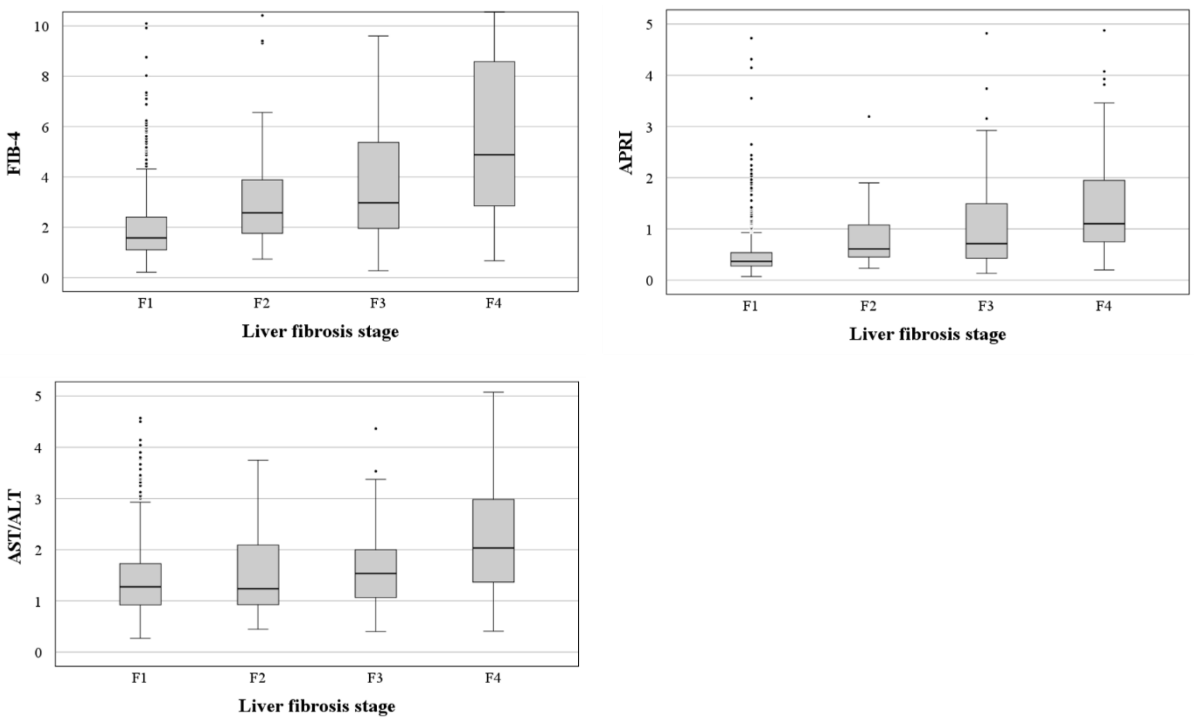

Supplementary Figure S2. Box plot of non-invasive hepatic fibrosis score. The gray box represents the interquartile range and the black line across the box indicates the median. “Whiskers” are black lines that extend from the box to the highest and lowest values, excluding outliers (dots).
